# Use of the Children’s Hospital of Philadelphia Infant Test of Neuromuscular Disorders (CHOP INTEND) in X-Linked Myotubular Myopathy: Content Validity and Psychometric Performance

**DOI:** 10.3233/JND-200479

**Published:** 2021-01-01

**Authors:** Tina Duong, Gale Harding, Sally Mannix, Cristina Abel, Dawn Phillips, Lindsay N. Alfano, Carsten G. Bönnemann, Charlotte Lilien, Linda P. Lowes, Laurent Servais, Birgit Warken-Madelung, Susie Nieto Bergman, Emma S. James, Mojtaba Noursalehi, Suyash Prasad, Salvador Rico, Deborah A. Bilder

**Affiliations:** a Stanford University, Palo Alto, CA, USA; b Evidera, Bethesda, MD, USA; c Nationwide Children’s Hospital, Columbus, OH, USA; d National Institute of Neurological Disorders and Stroke, Bethesda, MD, USA; e MDUK Oxford Neuromuscular Centre, Oxford, United Kingdom; f Institut I-Motion, Institut de Myologie, Paris, France; g University Hospital Liège & University of La Citadelle, Liège, Belgium; h Haunersches Children’s Hospital of the University of Munich, Munich, Germany; i Audentes Therapeutics, an Astellas Company, San Francisco, CA, USA; j Encoded Therapeutics, South San Francisco, CA, USA formerly at Audentes Therapeutics, an Astellas Company, San Francisco, CA, USA; k Suyash Prasad Consulting, LLC, San Francisco, CA, USA formerly at Audentes Therapeutics, an Astellas Company, San Francisco, CA, USA; i University of Utah, Salt Lake City, UT, USA

**Keywords:** XLMTM, CHOP INTEND, content validity, psychometric assessment, instrument, ASPIRO, INCEPTUS, resamirigene bilparvovec, gene therapy

## Abstract

X-linked myotubular myopathy (XLMTM) is a life-threatening, congenital myopathy characterized by extreme hypotonia, weakness, delayed motor milestones, and respiratory failure, often resulting in pediatric mortality. This study evaluated the content validity and psychometric performance of the Children’s Hospital of Philadelphia Infant Test of Neuromuscular Disorders as a measure of neuromuscular functioning in children with X-linked myotubular myopathy. This study was conducted in two phases. Phase I assessed the content validity of the measure for use in an XLMTM pediatric population through: literature review, clinical expert interviews, caregiver interviews, and a modified-Delphi panel among clinicians. Phase II assessed psychometric performance based on the INCEPTUS observational clinical study and the ASPIRO interventional gene therapy study, including tests of reliability (internal consistency, test-retest, and interrater), validity (construct and criterion), and responsiveness based on observational and interventional clinical trial data analyses. Data established construct validity and reliability of the Children’s Hospital of Philadelphia Infant Test of Neuromuscular Disorders among XLMTM patients before administration of resamirigene bilparvovec, and sensitivity to study drug administration as evidenced by the significant post-administration response in ASPIRO. Findings support the Children’s Hospital of Philadelphia Infant Test of Neuromuscular Disorders as an appropriate neuromuscular functioning assessment in a pediatric X-linked myotubular myopathy patient population.

## ABBREVIATIONS

BSID: Bayley Scales of Infant Development
CHOP INTEND: Children’s Hospital of Philadelphia Infant Test of Neuromuscular Disorders
MFM-20: Motor Function Measure-20
PRO: Patient-reported outcome
SMA: Spinal muscular atrophy
XLMTM: X-linked myotubular myopathy

## INTRODUCTION

X-linked myotubular myopathy (XLMTM) is a life-threatening, congenital myopathy, characterized by extreme hypotonia, weakness, delayed motor milestones, and respiratory failure, which often results in death in approximately half of infants before 18 months of age [1, 2]. According to the National Organization for Rare Disorders [3], XLMTM occurs in 1 of every 50,000 male births. The disease is caused by mutations in the *MTM1* gene, which is responsible for production of the myotubularin protein. Myotubularin is thought to play a role in the development, maintenance, and function of skeletal muscles [4, 5]. Although many children with this disease do not survive past infancy or early childhood, those who do often experience severe and debilitating symptoms, such as respiratory failure, difficulty or inability to swallow, paralysis of the eye muscles, hypotonia, gross motor dysfunction, and inability or extreme difficulty in communicating [3, 5]. Cognitive development is normal unless the central nervous system is compromised by severe hypoxic episodes [6]. Currently, there are no approved treatments for XLMTM.

Due to the severity of this disease and the range of effects on the entire body, children with this disease are almost always dependent on a power wheelchair for mobility, ventilator due to respiratory failure, gastrostomy feeding tube, and have a high rate of hospitalizations [1, 7]. In most patients, extreme muscle weakness results in an inability or severely delayed achievement of developmental motor milestones such as rolling, sitting, or standing, which necessitates dependency on caregivers for most activities of daily living and care [1, 7, 8]. Most acquired milestones are subsequently lost in severe phenotypes [8]. XLMTM carries a heavy burden of illness for both patients and caregivers [7].

Clinical assessments of function are increasingly used in the medical research community to assess disease prognosis and functional ability. They may serve as endpoints to determine potential treatment benefits related to motor skills that may impact patient quality of life [9]. A function-based clinical assessment is beneficial to patients and families because it allows healthcare professionals to measure function empirically to understand the natural disease course and possible treatment impact. In a disease such as XLMTM, the ability to measure function objectively is limited by profound muscle weakness. The severity of weakness requires the need for assistive devices, orthoses, and mobility and respiratory equipment. Most motor assessments commonly used to measure infant development (such as the Bayley Scales of Infant Development [BSID]) are insufficient to quantify functional motor skills in children with XLMTM because of the severity of their motor weakness that results in a propensity for the measurement to have a floor effect. Assessments that are more sensitive to small changes in motor function can improve clinician and families’ understanding of treatment response and better inform assistive device, respiratory needs, and other supportive or treatment recommendations.

In the context of clinical trials, instruments should be reliably performed and sensitive to changes that reflect the experiences of the patients. Regulatory agencies, such as the United States (US) Food and Drug Administration (FDA) and European Medicines Agency (EMA), are supportive of the incorporation of clinical trial outcomes using endpoints that capture how patients feel and function [10, 11]. When selecting these measures, there should be evidence of concept relevance to the target patient population; outcome measures should be validated in the target population.

The Children’s Hospital of Philadelphia Infant Test of Neuromuscular Disorders (CHOP INTEND) [12, 13] is a clinician-reported outcome (ClinRO) measure designed to assess motor function in very weak individuals with neuromuscular disorders and respiratory compromise. The repertoire of associated motor skills and movements can be quantified based on unelicited observation of upper and lower limb movement and elicited motor assessment of trunk, pelvis, and head functional strength without relying heavily on prone tasks that are more challenging for individuals with respiratory dysfunction. The scale contains 16 items, each of which is scored on a scale of 0–4. Based on a study of spinal muscular atrophy (SMA) and normal controls, a maximal score of 64 was typically obtained by 3–6 months in the normal controls [14]. Another study of SMA infants found a correlation between CHOP INTEND scores ≥50 and unassisted sitting [15].

The CHOP INTEND was originally designed to quantify motor abilities in infants aged 1.4–37.9 months with SMA type 1 (SMA-1) [12, 13]. Because of the similarities between SMA-1 and XLMTM disease symptomatology and neuromuscular involvement, the CHOP INTEND was selected as a clinical trial endpoint for the XLMTM pre-Phase 1 prospective, observational clinical study INCEPTUS.

In the INCEPTUS study, CHOP INTEND was evaluated as a measure of neuromuscular function in children with XLMTM by assessing the content validity and psychometric performance in this population. This study’s design was guided by best practice recommendations for patient-reported outcome (PRO) instrument content validation from the US FDA Guidance for Industry PRO Measures: Use in Medical Product Development to Support Labeling Claims [11], the International Society for Pharmacoeconomics, and Outcomes Research Task Force Reports [16, 17]. After validation of quantitative and qualitative findings from the INCEPTUS study to support its use in XLMTM, the CHOP INTEND was chosen as a co-primary endpoint in the ASPIRO gene therapy clinical trial for infants and children.

## MATERIALS AND METHODS

The research was conducted in 2 phases. Phase I addressed the content validity of the CHOP INTEND in an XLMTM pediatric population through qualitative research methods. Phase II addressed the quantitative properties of the measure, including tests of reliability (internal consistency, test-retest, and interrater), validity (construct and criterion), and responsiveness based on analyses of a non-interventional, observational clinical trial data from the observational clinical run-in study (INCEPTUS; NCT02704273), and assessment of responsiveness to change in an interventional clinical trial of the investigational gene therapy product resamirigene bilparvovec (formerly known as AT132) (ASPIRO; NCT03199469). Resamirigene bilparvovec is a non-replicating, genetically engineered adeno-associated virus gene therapy that expresses human myotubularin.

All data collection and recruitment procedures met institutional review board (IRB) and Health Insurance Portability and Accountability Act (HIPAA) requirements, as well as all applicable state and federal laws and regulations, with written informed consent obtained from study participants prior to completing any study-related activities.

### Phase I –Qualitative content validity assessment

**Fig. 1:**
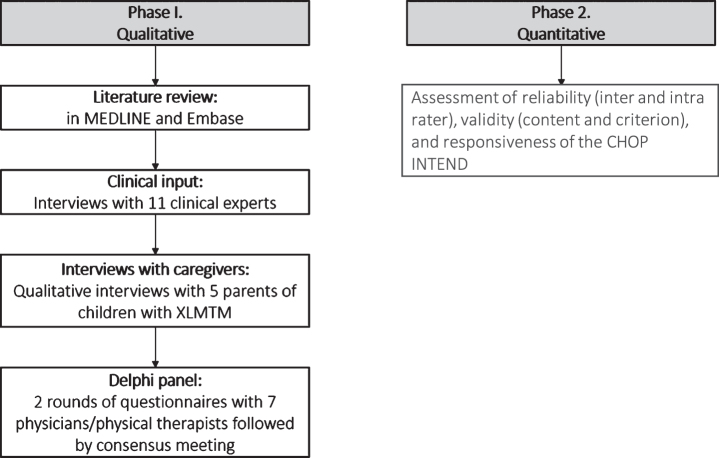
Flow diagram. Abbreviations: CHOP INTEND = Children’s Hospital of Philadelphia Infant Test of Neuromuscular Disorders; XLMTM = X-linked myotubular myopathy.

A multi-step qualitative research process was used to assess the content validity of the CHOP INTEND for use in a pediatric XLMTM population (Fig. 1). A review of the literature and interviews with therapeutic area clinical experts were conducted to cross-reference symptoms and inform the development of a conceptual model for XLMTM. Interviews with caregivers of patients with XLMTM were subsequently conducted to ensure the relevance of the CHOP INTEND for neuromuscular symptoms from the caregiver perspective. Finally, a Delphi approach among clinicians was used to finalize the conceptual model and confirm the relevance of the CHOP INTEND as an appropriate clinical assessment tool to assess neuromuscular function in a pediatric XLMTM population.

#### Literature review

A literature review was conducted to understand functional impacts of XLMTM in patients, with an emphasis on neuromuscular function. Two global biomedical literature databases were used, MEDLINE and Embase; search terms included myotubular myopathy; centronuclear myopathy; XLCNM; XLMTM; MTM1; MTM; symptom(s); impact(s); burden; quality of life; health-related quality of life; health status; movement; functional assessment; disease severity; weakness; disability; dysphagia; respiratory function; mechanical ventilator; hypoxia; asphyxia; dystonia; hypotonia; ambulation; walking; wheelchair/bedridden; and muscle strength/weakness. The search limits included human population, English language, articles with abstracts, and articles published since 1995.

#### Clinical input

Qualitative interviews were conducted in the US and European Union with 11 clinicians who have expertise working with children with XLMTM across 7 institutions located in 5 countries: the US (*n* = 5); France (*n* = 2); Germany (*n* = 2); Canada (*n* = 1); and the United Kingdom (*n* = 1). These experts comprised 6 physicians and 5 physical therapists from 7 institutions across 5 countries. The purpose was to define neuromuscular concepts of XLMTM and gain a better understanding of the relevant clinical outcomes related to functional abilities and body organ systems. The results of the expert interviews, in conjunction with the findings from the literature review, were used to develop a conceptual model of XLMTM and determine whether the CHOP INTEND is an appropriate assessment tool for neuromuscular function among pediatric patients with XLMTM. All interviews were conducted face-to-face in English, audio recorded, and transcribed for review and analysis.

#### Delphi panel

Commonly used in healthcare, Delphi methodology is an iterative process used to determine expert group consensus [18]. This study utilized a modified Delphi approach that consisted of 2 rounds of e-mail questionnaires and a final web-based teleconference to discuss the results and obtain consensus on the appropriateness of using the CHOP INTEND to assess neuromuscular function in pediatric XLMTM patients. While incorporating a final meeting was not part of the original Delphi method [19], the modified Delphi method includes this meeting to allow for expert interaction and to facilitate consensus [20].

Delphi methodology was used to synthesize the opinions regarding the use of the CHOP INTEND to assess motor function among pediatric XLMTM patients aged ≤5 years and to build consensus among the panel through a structured process that used a series of questionnaires and refined feedback.

**Fig. 2:**
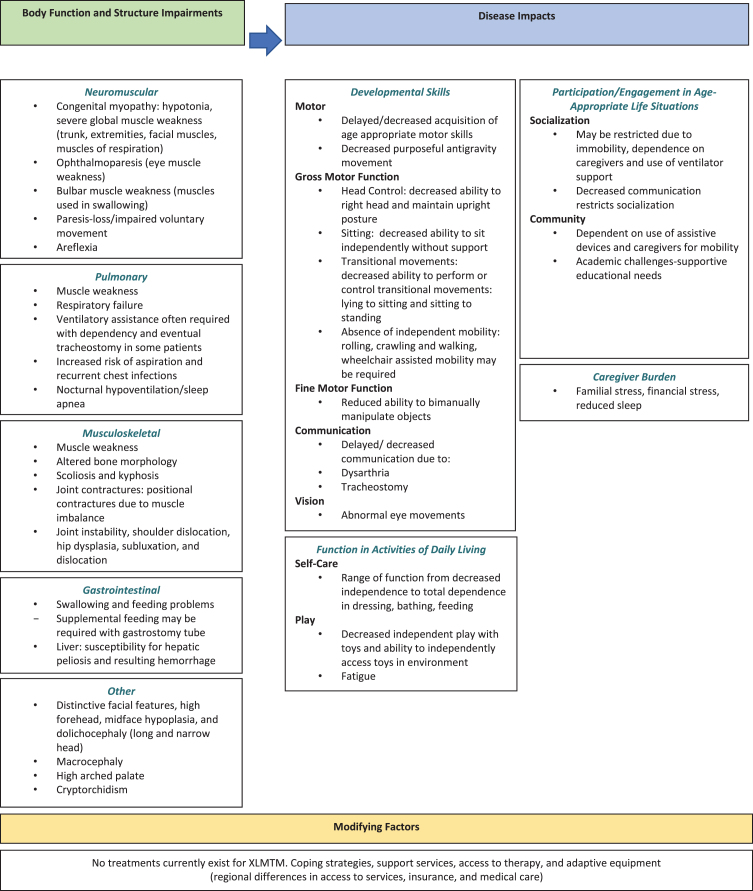
Conceptual model for XLMTM.

The Delphi panel consisted of 7 neuromuscular physicians and physical therapists from the US, Germany, France, Canada, and the United Kingdom who treat XLMTM and other neuromuscular disorders. Two rounds of questions that included feedback on a clinical conceptual model of XLMTM to determine areas of significant concerns in XLMTM (i.e., respiratory, speech, gross motor delays, hypotonia) (Fig. 2). This was followed by questions based on the clinical relevance and comprehensiveness of the CHOP INTEND for use in a clinical trial of pediatric patients with XLMTM. Results from Round 1 were compiled, anonymized, and included in the Round 2 survey to gain additional feedback, followed by a web-based teleconference to obtain final consensus on the conceptual framework and relevance of the CHOP INTEND for a XLMTM pediatric population.

#### Interviews with caregivers

One-on-one qualitative interviews with 5 parents of children with XLMTM were conducted to gain a better understanding of the symptom experience, burden, and humanistic impacts on daily life of patients and their caregivers. The interviews were conducted by telephone using a semi-structured interview guide; they were audio recorded for transcription and content analysis. Qualitative data from the interviews, including interviewer notes and/or audio transcriptions, were analyzed using a content analysis approach. Findings were used to further explore the relevance of CHOP INTEND as a measure of neuromuscular function from the perspective of the caregiver.

### Phase II –Quantitative assessment of reliability, validity, and responsiveness to change

The psychometric measurement properties of the CHOP INTEND, including reliability, validity, and responsiveness to change were evaluated among a stable sample of children with XLMTM aged <4 years at enrollment. ASPIRO participants were selected from INCEPTUS allowing for measurement of CHOP INTEND over time and its responsiveness to an intervention. Analyses were based on all available data. Form-level missing data were not imputed. Given the small sample size reflecting the rarity of XLMTM, analyses were not powered for statistical significance. However, significance levels of statistical tests are reported, and trends were examined. No adjustments were made for multiplicity. Statistical programming software SAS v9.4 was used for all statistical analyses.

#### Measures

The CHOP INTEND was conducted at baseline and every 3 months thereafter in all patients. The Motor Function Measure-20 (MFM-20) was conducted with the same frequency in all patients >2 years of age. Once a patient was able to sit independently or obtain *a* >45 score on the CHOP INTEND, the BSID was initiated.

All physical therapists performing the evaluations participated in two didactic training sessions at the start of the INCEPTUS and ASPIRO trials to decrease drift of knowledge and ensure consistency and standardization with the administration and scoring of the movement patterns seen in participants with XLMTM for all CHOP INTEND items. Missing values were not imputed. Prior to the initiation of the ASPIRO trial, reliability was measured by review of patient videos as part of the ASPIRO training. Respiratory function was also assessed by testing the maximum inspiratory pressure (MIP, also called P_I_max) and maximum expiratory pressure (MEP, also called P_E_max).

*MFM-20:* The MFM-20 includes 20 tests of functional muscle strength on a scale of 0–3, for a total score of 60. The questions capture 3 dimensions of muscle function: standing and transfers, axial and proximal motor function, and distal motor function. The MFM-20 has been validated in ambulatory and non-ambulatory patients aged 2–7 years with Duchenne muscular dystrophy, SMA, Charcot Marie Tooth, congenital myopathy, or congenital muscular dystrophy [21]. The MFM-20 was used to assess children ≥2 years of age.

*MIP and MEP:* MIP and MEP are estimates of respiratory muscle strength [22, 23]. MIP is a measure of diaphragmatic and inspiratory muscle strength, whereas MEP is primarily associated with expiratory muscle and cough strength, and thus ability to clear the airway.

#### Multi-method analytic approach

Three analytic approaches were used to examine the reliability, validity, and responsiveness of the CHOP INTEND in XLMTM patients, including regression analyses, correlational associations between concurrent measures, and analysis of variance (ANOVA) models. Given the small sample size and limited data, these approaches allowed all available data to be used in the models.

*Test-retest reliability*: Test-retest reliability reflects the ability of the instrument to provide reproducible results when the clinical state has been judged to be stable. To assess test-retest reliability, simple linear regression models were used to establish a data trend of the CHOP INTEND total scores over time by participants based on INCEPTUS observational study data. A non-significant *p*-value established stability (i.e., test-retest reliability). Pearson correlation analysis was also used to establish visit-to-visit stability (Table 1). A repeated-measures ANOVA model with visit (Months 0, 3, 6, 9, 12, 15, 18, 21, and early termination) as a fixed effect and participant as a random effect was used to further examine stability of the CHOP INTEND total score over time (Table 2). Similarly, a non-significant *p*-value established stability (i.e., test-retest).

**Table 1:** INCEPTUS—Pearson Correlations Between CHOP INTEND Assessments

	Pearson correlation			
	Screening	Month 3	Month 6	Month 9
Screening	1.00000	0.70412	0.93580	0.88760
Month 3	0.70412	1.00000	0.97286	0.92001
Month 6	0.93580	0.97286	1.00000	0.96056
Month 9	0.88760	0.92001	0.96056	1.00000

**Table 2:** INCEPTUS—Analysis of Variance Model for CHOP INTEND

Effect	LS Mean Estimate	SEM	Test	*P*-value^a^
Visit	—	—	0.59	0.7832
Month 0	33.3684	2.0251
Month 3	35.6154	2.4483
Month 6	36.1000	2.7915
Month 9	31.0000	2.5482
Month 12	30.2222	2.9425
Month 15	32.8000	3.9477
Month 18	36.6000	3.9477
Month 21	35.0000	8.8274
Early termination	32.2727	2.6616

^a^Mixed effect model with repeated/random effect and visit as fixed effect. Abbreviations: LS = least square; SEM = standard error of measurement.

*Inter-rater reliability:* Inter-rater reliability reflects the level of agreement that is present among responses when a measure is administered by two or more raters on the same individual. An inter-rater reliability analysis was completed as part of the CHOP INTEND rater training for the ASPIRO clinical trial. Fifteen physical therapists experienced with assessing XLMTM patients clinically and as a part of the INCEPTUS study, rated all items on the CHOP INTEND through observation of a video in which a patient with XLMTM was evaluated using the instrument. Training through video observation was chosen because of the fragility and fatigability of patients with XLMTM. During the training, each rater was unaware of the scores of the other raters. Pearson correlations and Cronbach *α* general scores for association were examined between the raters to assess inter-rater reliability. A Cronbach *α* score >0.85 was deemed acceptable, and >0.90 was interpreted as excellent.

*Validity:* Construct validity is the extent to which an instrument measures the construct(s) it is intended to measure, based on examining correlations with other indicators of similar/related concepts [24]. The strength of associations between CHOP INTEND total scores and other neuromuscular and respiratory parameters MFM-20, MIP, and MEP were examined across all visits using Pearson correlation, Cronbach *α* score, and regression model (Table 3). No specific hypotheses were made with respect to the magnitude of correlations. However, a higher correlation, Cronbach *α*>  0.80, and *p*-value <0.10 were used as a general guide to establish strength of association between the CHOP INTEND and other measures.

**Table 3:** INCEPTUS—Correlation Matrix Between CHOP INTEND and Other Efficacy Parameters

	Pearson correlation			
	CHOP INTEND	MFM-20	MIP	MEP
CHOP INTEND	1.00000	0.83601	0.46231	0.39964
MFM-20	0.83601	1.00000	0.41152	0.42240
MIP	0.46231	0.41152	1.00000	0.83068
MEP	0.39964	0.42240	0.83068	1.00000
	Cronbach Coefficient Alpha				
	Raw: 0.8431				
	Standardized: 0.8361				

Abbreviations: CHOP INTEND = Children’s Hospital of Philadelphia Infant Test of Neuromuscular Disorders; MEP = maximum expiratory pressure; MFM-20 = Motor Function Measure-20; MIP = maximum inspiratory pressure.

*Responsiveness:* To examine sensitivity and responsiveness to change of the CHOP INTEND, we used the ASPIRO Phase 1/2 interventional clinical trial. INCEPTUS data (regression, correlation, Cronbach *α*, and ANOVA model analyses) were used to show stability prior to drug administration in the ASPIRO trial, the time course of CHOP INTEND total scores over time was plotted using INCEPTUS and ASPIRO (post-study drug administration data) for all participants in ASPIRO to provide visual and statistical (locally estimated scatterplot smoothing [LOESS] regression model) evidence of efficacy response to study drug. At the time of this analysis, only 2 participants had reached 36 weeks in the ASPIRO study.

## RESULTS

### Phase I –Qualitative content validity assessment

#### Literature review

A total of 16 observational and case studies were reviewed [2, 25–39], which underscores the paucity of research in XLMTM due to the rareness of the condition. Findings indicate that hypotonia is a severe neuromuscular consequence among patients with XLMTM with significantly impaired respiratory function, usually resulting in the need for ventilator support [25, 29, 32, 34, 35, 38, 39]. Most patients with XLMTM do not obtain ability to walk [2, 8, 25, 26, 28, 30, 32, 34, 35]. In a natural history study of 50 patients with XLMTM, only 13% achieved the ability to stand or walk while 52% achieved the ability to sit independently as their highest milestone [7]. The most recent prospective international natural history study shows that most patients had severe delays in motor acquisition followed by progressive loss [8]. Patients often experience ocular dysfunction [26, 28, 29, 33, 35, 39], such as ophthalmoplegia [29, 33], as well as facial weakness [28, 37–39], scoliosis [30, 36–38], orthopedic manifestations [26, 30, 32, 36], gastrointestinal malfunction [32, 35, 39], and renal complications [27, 32]. Assessments of neuromuscular function described in the articles were limited, with several studies using non-specific questionnaires completed by families and/or clinicians [2, 34, 36, 37].

#### Clinical input

Common important clinical themes were consistent with the literature review surrounding neuromuscular concepts (*n* = 7) and respiratory functioning (*n* = 7). The neuromuscular concepts included gross motor function and strength, feeding/swallowing, ambulation, head/neck functioning, fine motor functioning, weakness, and lack of volitional movement. Following these were communication (*n* = 3), reaching age-appropriate milestones (*n* = 2), and ophthalmology (*n* = 2). Themes mentioned once included cognitive status, ability to engage with environment (requiring muscle strength and motor skills), and quality of life.

When the experts were asked about aspects of XLMTM that are most important to include in an assessment, neuromuscular concepts—specifically, those related to strength, motor functioning, and movement—were the most prominent (*n* = 9). Key impairments reported by the clinicians included concerns with strength, motor function, joint range of motion, anti-gravity strength in extremities (lifting legs, arms, elbows) and associated functional difficulties with gross motor development, upper and lower limb functions, head control, and trunk control/sitting. Based on the clinical description and understanding of XLMTM, many of the clinicians believed that even though the CHOP INTEND was not designed to capture motor milestones, it was the most appropriate scale based on the clinical presentation of XLMTM to capture the degree of minimal motor movements demonstrated by most individuals with XLMTM. Other motor scales such as the BSID were recommended to be introduced if patients were able to sit independently or scored >45 on the CHOP INTEND.

#### Interviews with caregivers

Five caregivers of male children with XLMTM were interviewed. The children with XLMTM ranged in age from 20 months to 4 years, with all caregivers reporting that their child had moderate to very severe disease.

All caregivers (5/5; 100%) reported that their children experienced general muscle weakness. All but 1 caregiver (4/5; 80%) reported that their child experienced impaired gross motor function, eye problems (i.e., lack of blinking, dryness), and distinctive facial characteristics common to children with XLMTM. Breathing problems, including respiratory infections and dysphagia, were also reported (3/5; 60%). All children (100%) were ventilator-dependent, and management of the ventilator and respiratory health was commonly mentioned. Developmental milestones were generally delayed or not reached. All caregivers reported that XLMTM impacted their activities of daily living and quality of life. The most impactful effects of XLMTM on the child were reported to be respiratory-related (5/5; 100%) and immobility (2/5; 40%).

The responses provided by the caregivers regarding the function of their child strongly aligned with the motor functional abilities assessed in the CHOP INTEND. Based on descriptions provided by caregivers during their interviews, all 5 caregivers (100%) endorsed concepts related to decreased voluntary active upper extremity movement/strength. These concepts map to CHOP INTEND item 1 (spontaneous movement, upper body), item 8 (shoulder and elbow flexion and horizontal abduction), item 9 (shoulder flexion and elbow flexion), and item 13 (elbow flexion). Similarly, all caregivers indicated concerns with concepts related to decreased voluntary active lower extremity movement/strength, with these concepts mapping to CHOP INTEND item 2 (spontaneous movement, lower extremity), item 5 (hip adductors), item 10 (knee extension), and item 11 (hip flexion and foot dorsiflexion). All caregivers indicated concerns with concepts related to grasping, mapping to CHOP INTEND item 3 (hand grip). Additionally, all 5 caregivers indicated concerns with concepts related to decreased trunk strength and head control. These concepts map to CHOP INTEND item 4 (head in midline with visual stimulation), item 12 (head control), item 14 (neck flexion), item 15 (head and neck extension, Landau), and item 16 (spinal incurvation, Galant). Finally, 4 caregivers (80%) indicated concerns with concepts related to decreased mobility, which relate to CHOP INTEND item 6 (rolling elicited from legs) and 7 (rolling elicited from arms).

Findings from the literature review and clinical and caregiver interviews informed the initial conceptual model for XLMTM, which provided initial evidence that the CHOP INTEND covered most key concepts related to motor function for individuals with XLMTM.

#### Delphi panel

Findings from Round 1 indicated that the conceptual model needed to be reorganized to incorporate the structure of the International Classification of Function, Disability and Health—World Health Organization (ICF-WHO) model (Fig. 2). With respect to the CHOP INTEND, key advantages identified in Round 1 included 1) the ability to measure early small changes; 2) the focus on basic motor function; and 3) the instrument is widely known, reproducible, and easy for clinicians to learn and perform. An area of concern regarding the use of the CHOP INTEND included challenges in completing 3 CHOP INTEND items for larger and/or heavier children, specifically item 11 (hip flexion and foot dorsiflexion, measuring anti-gravity leg movement), item 15 (head and neck extension, measuring health and neck lift against gravity), and item 16 (spinal incurvation, measuring isolated trunk activity in response to a stimulus or weight shift). These items require the evaluator to lift the patient, making assessments more difficult with safety concerns for older/heavier children. The Pediatric Neuromuscular Clinical Research (PNCR) Network for SMA includes instruction in the CHOP INTEND manual on recommended strategies for administration for older children who are no longer infants. This manual describes adaptations for larger children for these items [40]. The instructions for administration of CHOP INTEND items 11, 15, and 16 were clarified, based on these recommendations, to address assessment of larger children, and included in the Round 2 materials for comment from the Delphi Panel members.

Findings from Round 2 indicate that there was consensus on the revised conceptual model. While panel members favored the recommended clarifications to the 3 CHOP INTEND items (11, 15, 16), several panelists had additional recommendations and felt that the items would still be challenging for larger/heavier children. These issues were further discussed during the web-based teleconference, with the panel agreeing that it was important to include these items to retain the integrity of the instrument. The deletion of these higher-level items may increase the ceiling effect on the total score. The Delphi panel reached consensus that the CHOP INTEND is appropriate for patients aged ≤5 years with XLMTM. Administration training focused on highlighting options to accurately adapt the item to variable patient sizes and functional levels without changing the construct of the item.

### Phase II –Quantitative assessment of reliability and validity

**Fig. 3:**
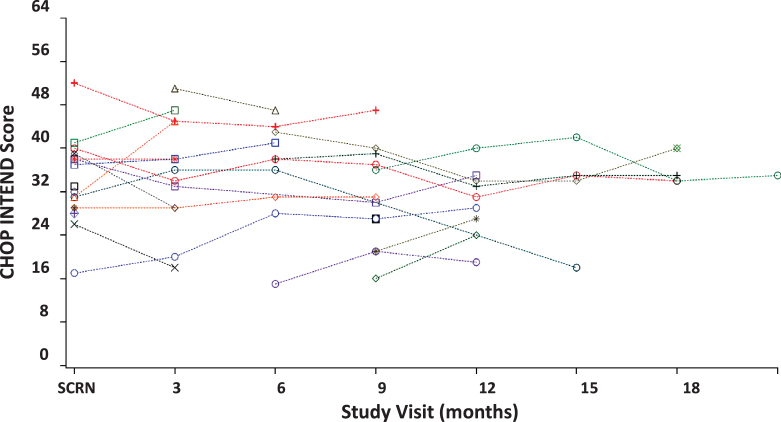
INCEPTUS—CHOP INTEND assessment scores over time (N = 28). Each symbol and dotted line represents a patient; CHOP INTEND = Children’s Hospital of Philadelphia Infant Test of Neuromuscular Disorder; Abbreviation SCRN = screening.

Clinical characteristics for the study sample are included in Table 4. CHOP INTEND data from the INCEPTUS observational clinical study demonstrate that all participants have severely impaired motor abilities. The majority of the scores fall within the 30- to 40-point range (Fig. 3), which is consistent with SMA [14, 41] and XLMTM [8] natural history studies and indicates profound neuromuscular impairment. BSID was initiated only when a score of 45 was obtained on the CHOP INTEND or participants gained the ability to sit unassisted. At the time of this analysis, insufficient data were available and therefore the BSID is not included in this study.

**Table 4:** Baseline Characteristics

Study characteristics	Baseline characteristics	
	INCEPTUS (*N* = 28)	ASPIRO (*N* = 6)
Age (years)
N	28	6
Mean (SD)	1.8 (1.14)	1.6 (1.42)
Median	1.6	0.8
Range	0.3, 4.6	0.7, 4.1
Missing	0	0
Randomized control participants*	—	3.9
CHOP INTEND total score
N	19	6
Mean (SD)	33.4 (7.65)	37.7 (5.92)
Median	32.0	37.5
Range	17.0, 52.0	29.0, 45.0
Missing	9	0
Randomized control participants*	—	49.0
MIP (cmH_2_O)
N	18	6
Mean (SD)	25.0 (14.52)	30.0 (10.03)
Median	19.0	30.9
Range	7.0, 58.7	14.1, 44.1
Missing	10	0
Randomized control participants*	—	58.1
MEP (cmH_2_O)
N	18	6
Mean (SD)	25.0 (14.52)	22.6 (8.06)
Median	19.0	20.8
Range	7.0, 58.7	13.9, 34.9
Missing	10	0
Randomized control participants*	—	31.5

*ASPIRO study only; Delayed treatment randomized control participants, not yet treated, N = 1. Abbreviations: CHOP INTEND = Children’s Hospital of Philadelphia Infant Test of Neuromuscular Disorders; cmH_2_O = centimeters of water column; MEP = maximum expiratory pressure; MIP = maximum inspiratory pressure; SD = standard deviation.

#### Inter-rater reliability

All correlation values between raters for the individual CHOP INTEND items were above 0.70, with many near 1.0000. Cronbach *α* was calculated to be 0.9956, confirmation of a strong inter-rater reliability of the instrument.

#### Validity

Correlations between the CHOP INTEND total scores and the assessed neuromuscular and respiratory parameters MFM-20, MIP, and MEP across all INCEPTUS visits are presented in Table 3. Results indicate a high correlation of the two functional strength assessments between CHOP INTEND total scores and the MFM-20 total scores (*r* = 0.84), demonstrating excellent construct validity. Correlations with clinical parameters of respiratory muscle strength were moderate (0.46 for MIP; 0.40 for MEP), demonstrating criterion-related validity. Not surprisingly, the strength of the correlation is higher between the two scales with similar motor construct (CHOP INTEND and MFM-20). The weaker, though still significant, correlation between the CHOP INTEND and assessments of respiratory muscle strength demonstrate the importance and complementary aspects of measuring both concepts concurrently.

#### Test-retest reliability

Figure 3 depicts participants’ CHOP INTEND assessment scores for up to 21 months during the INCEPTUS observational clinical study. When examining the regression line fitted to all INCEPTUS patients over all assessments, the slope was small in magnitude, slightly declining, and not statistically significant (slope = –0.070, *p* = 0.5578), showing stability of the scores across visits and patients, as well as reflecting the slow clinical deterioration of patients during the period of observation (Table 5).

**Table 5:** INCEPTUS—Regression Model Across Visits

	Variable	Estimate	SEM	Test	*P*-value
All visits	Intercept	34.02709	1.42238	23.92	<0.0001
	Slope	–0.06968	0.11840	–0.59	0.5578

Abbreviation: SEM = standard error of measurement.

In INCEPTUS, the correlations between CHOP INTEND total scores across visits were high and significant, ranging from 0.70 to 0.94 (Table 1), suggesting that reliability and stability improved with time. The improved stability was evident by the significant and tight range for correlations across visits at 3, 6, and 9 months following screening, ranging from 0.92 to 0.97. The generalizability coefficient (Cronbach *α*) across assessment visits using all measurements was high (*r* = 0.97) further supporting the test-retest stability as well as intra-rater reliability of the instrument as the same physical therapist performed the assessments for each patient. In addition, the least squares mean estimates for visits between screening and Month 21/early termination did not significantly differ (*p* = 0.7832; Table 2), indicating stability of scores and minimal change in clinical symptomatology over time.

#### Responsiveness

**Fig. 4:**
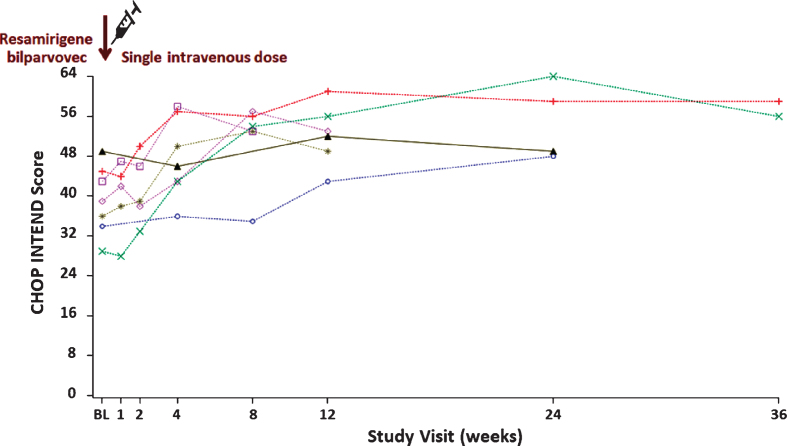
ASPIRO—CHOP INTEND assessment scores over time (N = 6 dosed, N = 1 untreated control). Abbreviation: BL = baseline; CHOP INTEND = Children’s Hospital of Philadelphia Infant Test of Neuromuscular Disorders. Each symbol with dotted line represents a patient who received resamirigene bilparvovec (N = 6), and the solid brown triangles with solid line represents the untreated control patient (N = 1).

**Fig. 5:**
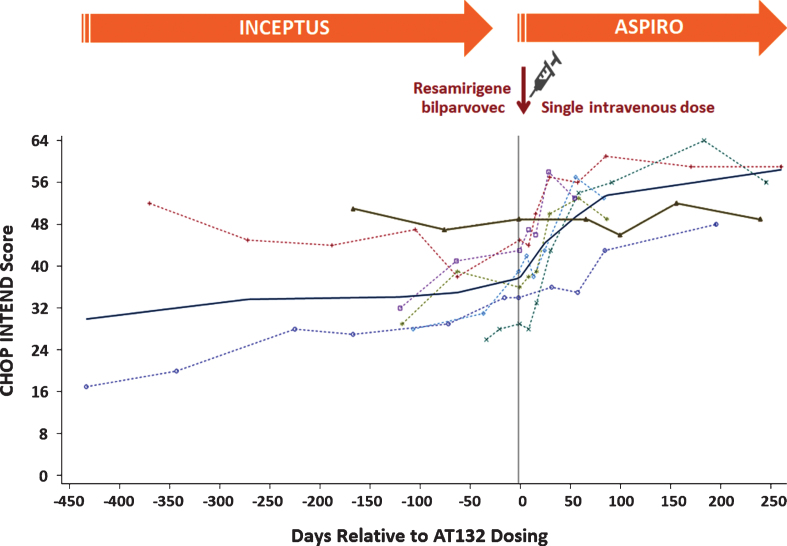
INCEPTUS and ASPIRO—CHOP INTEND assessment scores over time. Abbreviations: CHOP INTEND = Children’s Hospital of Philadelphia Infant Test of Neuromuscular Disorders. Each symbol and dotted line represents a patient who received resamirigene bilparvovec, and the solid brown triangles with solid line represents the untreated control patient. The solid dark blue line represents the smoothed LOESS regression line fitted to the entire data for patients dosed with resamirigene bilparvovec, depicting the overall trend in the data over time for the dosed patients (20 July 2018 datacut).

Figure 4 displays the CHOP INTEND total score for all participants up to 36 weeks of treatment in ASPIRO, including randomized delayed-treatment control participants (prior to treatment). An increase in slope was seen starting 2 weeks after dosing of resamirigene bilparvovec, whereas the control participants continued the same pattern as in the INCEPTUS study, with no change from baseline (Figs. 4, 5). The CHOP INTEND regression line was positive and statistically significant, with a weekly slope change of 0.61 (*p* <  0.0001) after a single dose of resamirigene bilparvovec (Table 6).

**Table 6:** ASPIRO—Regression Model for CHOP INTEND Across Visits

	Variable	Estimate	SEM	Test	*P*-value
All visits	Intercept	42.46358	1.50111	28.29	<0.0001
	Slope	0.56353	0.12275	4.59	<0.0001
All treated participants (*n* = 6)	Intercept	42.00644	1.60384	26.19	<0.0001
	Slope	0.61084	0.13277	4.60	<0.0001

Abbreviation: SEM = standard error of measurement.

Figure 5 displays CHOP INTEND data for all treated participants from enrollment in INCEPTUS through their last post-treatment evaluation in ASPIRO. The solid dark bold and blue line in the figure represents the smoothed LOESS regression line fitted to the entire data for dosed participants, depicting the overall trend in data over time showing a mean rise in CHOP INTEND scores after dosing.

## DISCUSSION

In rare diseases where there is a lack of validated clinical outcomes to measure the natural history of the disease, FDA guidance notes that when well-defined clinical assessments are unavailable, “sponsors should recognize the need to develop new assessment tools, or modify existing ones, early to maximize time to develop and evaluate a new tool before relying upon it as the basis of an endpoint in a clinical trial” [42]. Among the substantial challenges needed to develop new, fully validated instruments to assess clinically meaningful endpoint, the FDA guidance highlights key characteristics, including validity, reliability, feasibility, resistance to bias, ability to detect change, relationship to meaningful symptoms or function, and clinical interpretability [42].

The current study was conducted to assess the feasibility and psychometric properties of the CHOP INTEND as an appropriate measure of neuromuscular function among patients with XLMTM. Originally validated for use with the SMA-1 population, the CHOP INTEND has since been validated for use in multiple neuromuscular diseases, including infant botulism, nemaline myopathy, and central core myopathy, all of which have a similar phenotype of extreme muscle weakness, hypotonia, impaired respiratory status, and a high likelihood of assisted ventilation [12, 13, 43–47]. Functional baseline characteristics were similar to the XLMTM INCEPTUS cohort including inability to obtain or severely delayed acquisition of motor milestones such as rolling, sitting, or standing [45–47].

Symptoms and impact on neuromuscular function described by clinicians and caregivers strongly align with the XLMTM literature on phenotypical presentation and progression that may be assessed using the CHOP INTEND. The INCEPTUS study supported the CHOP INTEND’s use as a sound measurement tool with excellent reliability, validity, and stability across the 21 months of the observational clinical study. INCEPTUS baseline CHOP INTEND scores were similar to baseline measures of other SMA-1 and XLMTM natural history studies [8, 14, 41]. In regards to responsiveness, the INCEPTUS slope change of –0.070 indicates a fairly stable untreated cohort. The rapid weekly rise in slope of 0.61 after dosing in the ASPIRO trial, which is nearly 9× greater than the previous 21 months of evaluation, indicates that the CHOP INTEND is sensitive and responsive to change.

Additionally, the CHOP INTEND was a secondary endpoint in the ENDEAR study, a randomized, double-blind, sham-controlled, Phase 3 clinical trial of nusinersen in infants with SMA-1 (NCT02193074). A clinically meaningful CHOP INTEND response was defined as an increase of at least 4 points from baseline in CHOP INTEND score at the end-of-trial visit. This endpoint was achieved in 71% of the nusinersen-treated patients vs. in 3% of the control group (*p* <  0.0001) [48]. The CHOP INTEND was also an endpoint in a gene therapy study for SMA-1 (NCT02122952). Results demonstrated a rapid increase from baseline in the CHOP INTEND score, with an increase of 15.4 points at 3 months following a single systemic administration of scAAV9.CB.hSMN, compared with a decline in this score in a historical cohort [49].

This research is limited by small sample sizes and ceiling effect of the CHOP INTEND during the ASPIRO interventional trial. The CHOP INTEND demonstrated suitability in an untreated XLMTM participants in INCEPTUS, who had profound hyptonia, weakness, and lacked significant motor improvements that would otherwise result in a significant floor effect with other neuromotor measures. This floor effect of other measures was observed in a natural history study of very weak individuals with SMA who were not able to sit independently. The highest score on the Hammersmith Functional Motor Scale Expanded (HFMSE) was 2, lacking sensitivity to track subtle changes over time [50]. In the study of SMA-1 and normal controls with a mean age of 3 months, SMA-1 patients averaged 21 points on the CHOP INTEND compared with 50 points for controls [14]. In the Nusinersen trial, no untreated SMA-1 patient scored above 40; however there was a need post-treatment to transition to a scale that measures higher motor function [51]. Once patients begin achieving motor milestones (i.e., independent rolling or sitting), typically scoring ≥50 points [15] on the CHOP INTEND, a plateau in scores has been noted due to participants refusing to stay in the supine/sidelying position or having outgrown the motor tasks [48]. Because the CHOP INTEND was originally designed for infants, some items are based on primitive reflexes such as grip, Galant, and Landau reflexes. For older children, these reflexes would be expected to become integrated into voluntary motor control. The effort-dependent aspect of these movements requires the child’s cooperation and may subsequently impact the ability to assess these motor skills. Although arousal states were captured with the Brazelton Neonatal Behavior Assessment at administration of the CHOP INTEND in this study, arousal states are not synonymous with cooperation, particularly in children with higher capacity for voluntary movement.

Patients treated with resamirigene bilparvovec have the potential to reach the maximum score of 64 points and thus may register no further improvements on the CHOP INTEND scale despite continuing to improve in their physical functionality as indicated by the MFM-20 and BSID. A limitation of this study was timing of other assessments. Because the BSID was not performed in parallel with the CHOP INTEND, emerging motor milestones were not captured alongside the CHOP INTEND. Instead, the BSID was performed after the patient obtained a score of >45 on the CHOP INTEND (or was able to sit independently).

## CONCLUSIONS

Results from this study demonstrate that the CHOP INTEND is an appropriate, reliable, and valid clinical assessment tool of neuromuscular function in an untreated pediatric patient population with XLMTM. The CHOP INTEND has a ceiling effect for patients who gain higher motor skills such as the ability to sit, requiring additional clinical outcome measures to capture emerging higher motor abilities and milestones over a longitudinal assessment of treated patients. The authors gratefully acknowledge the following patient advocacy organizations for their assistance with recruitment of participants for the caregiver interviews: Joshua Frase Foundation and MTM-CNM Family Connection and patients who participated in this study. Becky Norquist provided medical writing assistance on behalf of Audentes Therapeutics.

## FUNDING

Funding for this project was provided by Audentes Therapeutics (San Francisco, CA).

## CONFLICT OF INTEREST

The SNB, EJ, MN, SP, and SR are or were contractors and employees of Audentes Therapeutics. GH, SM, CA, and DP are salaried employees of Evidera and are not allowed to accept remuneration from any clients for their services.
